# Supplementary material for: Are carbon emissions trading and green financial instruments synergistic? -Comprehensive quantitative research based on content analysis
Source: PLoS One. 2024 Mar 7;19(3):e0298601. doi: 10.1371/journal.pone.0298601 (PMC10919601; doi:10.1371/journal.pone.0298601)
Supplement: S1 Data — (ZIP) [file pone.0298601.s001.zip › Data/地级市Z_F工作报告.zip!/╕ú╜¿/┴·╤╥╩╨/└┤╫╘╣1⁄2╓┌║┼í╛┬φ┐╦╩2╛▌═°í┐_2013.docx]

各位代表：

　　现在，我代表市人民政府向大会作工作报告，请予审议，并请市政协委员和各位列席人员提出意见。

　　一、2012年工作回顾

　　过去一年，在市委的正确领导下，全市各级政府坚持以科学发展观为统领，围绕建设“五个龙岩”，牢牢把握“稳中求进”总基调，积极应对严峻复杂经济形势，全市经济社会呈现稳中有进的发展势头，较好实现了“三个高于”目标。初步统计，全市实现地区生产总值1375亿元，增长12%；财政总收入237.3亿元、增长15%，地方财政收入101.5亿元、增长20.4%；全社会固定资产投资1000.5亿元，增长28.5%；城镇居民人均可支配收入23765元、增长12.7%，农民人均纯收入9396元、增长14.1%。

　　1．农业生产平稳发展，农村建设扎实推进。实现农林牧渔业总产值265.8亿元，增长3.7%。粮食总产稳步增加，烤烟、茶叶、果蔬等产业加快发展。农产品加工业产值155.4亿元，增长19.1%。新发展农民专业合作社330家。新罗森宝集团成功创建全省首个国家级农业企业技术中心。完成水利建设投资20.4亿元，烟草水源工程、土地整理、病险水库除险加固工程顺利实施，解决42万农民饮水安全问题。“造福工程”搬迁1.6万人。启动“三农”综合示范点建设，成功举办首届农业展览会。“三农”综合保险取得新成效，上杭成为国家现代农业示范区，永定、长汀、漳平被列入全国新型职业农民培育试点县。

　　2．工业经济较快增长，产业支撑作用增强。出台扶持工业稳定增长12条政策措施，开展“千名干部进千企”帮扶活动。实现规模以上工业增加值446.8亿元，增长17.1%。"1+2"主导产业和"155"产业基地发展壮大，其中有色金属产业实现产值213.4亿元、增长46%，10个工业产业基地实现产值1030亿元、增长14.3%。稀土、硬质合金、光电、新材料等新兴产业加快发展。龙岩经济开发区、稀土工业园区分别升级为国家级经济技术开发区、省级工业园区，成功创建省级高新技术产业开发区。新上规模以上工业企业41家、累计1019家。龙洲运输、漳平木村成功上市。

　　3．第三产业稳步提升，旅游金融实现新突破。社会消费品零售总额423.5亿元，增长15.5%。新增限额以上商贸企业126家、累计691家，贸易业商品交易额210亿元、增长9.5%。陆地港保税物流中心、美食城一期等投入运营，中外运综合物流中心、龙门物流园二期等项目开工建设。旅游总收入108亿元、增长22.2%，接待游客1480万人次、增长22%。志高神州欢乐园顺利开园，全市新增3个国家4A级旅游景区。金融机构本外币存、贷款余额双双突破千亿元大关。民生、交通银行入驻龙岩。房地产开发投资120.5亿元，增长37.1%。

　　4．“五大战役”深入实施，重大项目顺利推进。超额完成"8432"计划，重点项目完成投资503.1亿元、增长35.2%。新龙马年产15万辆微车、紫金20万吨铜冶炼、瓮福紫金磷化工、龙化异地技改扩建一期、天守超纤纺织一期等项目投产，漳平现代特钢铸造基地、赣龙铁路扩能改造工程等项目加快建设，年产30万台发动机和西气东输三线工程、海西天然气管网、漳永高速龙岩段开工建设。龙厦铁路动车开通，双永、蛟城高速建成通车，实现县县通高速公路。

　　5．“城市建设年”工作成效显著，城镇化进程加快。认真落实市委“城市建设年”工作部署，开展中心城市千人大会战，组织实施九大片区改造，创新建设投融资、房屋土地征收、部门联动执法、市区工作等体制机制，完成征地5345亩，征收房屋124.3万平方米。石锣鼓湿地公园等项目基本建成，外环路和一批城市综合体加快建设。“五城同创”有序推进，成功创建国家园林城市。长汀、连城等县城市规划水平提升，旧城改造和新区开发力度加大，城市框架进一步拓展，城市特色更加凸显。15个省、市小城镇综合改革建设试点完成投资156.7亿元。全市城镇化率达50%，同比提高2.5个百分点。

　　6．生态建设全面加强，环境质量不断改善。深入贯彻习近平总书记的重要批示精神，完成水土流失治理48.6万亩、占省下达任务的150.6%。植树造林44.1万亩，占省下达任务的116%。长汀水土流失治理经验在全国推广，被评为全国生态文明和现代林业建设示范县。新罗万安、漳平永福获国家级生态乡镇命名。稳步推进龙津河流域综合治理，关闭落后产能企业58家。预计单位GDP综合能耗下降5.6%，主要污染物减排完成省下达任务。中心城市空气环境质量进一步提升。

　　7．改革开放不断深化，发展活力持续增强。全面启动“三区三园”体制创新和规划建设。农村耕地流转比例居全省首位，武平在全省率先启动农村集体土地抵押贷款。参照执行西部地区政策新增10项、累计76项，获得补助49.7亿元、累计106.4亿元。成功举办千名企业家大会、第三届海峡两岸机械产业博览会。对接“三维”项目397个，完成投资320.3亿元。实际利用外资8.6亿美元，增长22%；内联引资实际利用市外资金141.9亿元，增长10.6%。外贸出口总值21.1亿美元、增长13.9%，连续两年被评为全国外贸百强城市。民营经济加快发展，民间投资占全社会固定资产投资的三分之二。成功接待塔吉克斯坦总统来岩访问。新罗区入选全国百强区。永定客家文化园被授予全国“海峡两岸交流基地”。

　　8．民生保障更加有力，和谐社会建设取得新成效。35项为民办实事项目全面完成。新增城镇就业2.4万人、农村劳动力转移就业5万人，城镇登记失业率2.5%。最低工资、农村低保、新农合人均筹资标准和城乡居民基础养老金水平进一步提升，生育、失业、医疗保险参保覆盖面扩大，工伤保险实现全覆盖，城乡低保、困难家庭救助和养老服务力度加大，新型农村和城镇居民社会养老保险工作受国务院表彰。全市保障性住房开工建设1.4万套。新建、改扩建公办幼儿园33所，完成义务教育标准化学校评估178所，校安工程建成10.4万平方米，城区中小学扩容工程加快推进，龙岩学院通过教育部本科教学评估。乡镇卫生院“百院建设、千人培训”计划和510所村级卫生所建设稳步推进，市中医院病房大楼、第三医院门诊综合大楼投入使用，市妇幼保健院、儿童医院开工建设。成功承办福建省苏维埃政府成立80周年纪念大会、省第五届艺术节暨第25届戏剧会演等活动，农家书屋实现行政村全覆盖。广播电视“村村通”提升工程顺利实施。人口计生工作均衡发展。“菜篮子”工程加快实施。食品药品监管力度加大。“平安龙岩”建设扎实推进，信访积案化解工作走在全省前列。安全生产“四项指数”连续七年全面下降。村级组织换届全面完成。军政军民团结巩固发展，龙岩和连城、上杭获得全国“双拥模范城（县）”。人事、统计、民族宗教、外事侨务、老区扶贫、优抚安置、库区移民、对口援建、气象、地震、档案、地方志、邮政、老年人、妇女儿童、青少年、科普、残疾人等工作取得新进展。

　　9．自身建设进一步加强，政府运作效能提高。自觉接受市人大及其常委会的法律监督、工作监督和市政协的民主监督，广泛接受社会监督。交由政府系统承办的人大代表建议和市政协提案全部办结。依法行政和“六五”普法工作扎实开展。电子政务和政府信息公开深入推进，建成全省首个政务信息网桌面云系统。绩效管理更加科学，廉政建设、行政监察、审计监督和纠风工作进一步加强。

　　各位代表，过去一年我们所取得的成绩来之不易，这是市委正确领导的结果，是全市人民齐心协力、奋力拼搏的结果，是社会各界广泛参与、大力支持的结果。在此，我代表市人民政府，向全市广大干部群众，向人大代表、政协委员、各民主党派、工商联、人民团体、无党派人士、离退休老同志和社会各界人士，向中央和省直驻岩机构、驻岩部队、武警官兵、公安政法干警，向所有关心支持龙岩发展的港澳台同胞、海外侨胞、国际友人，表示衷心的感谢和崇高的敬意！

　　我们也清醒地认识到存在的问题与不足。主要是：经济总量小，产业层次低、结构不合理，科技创新能力弱；城镇化水平不高；生产性大项目少；出口竞争力不强，民营经济发展不够充分；生态环境保护压力较大；基本公共服务还有差距，安全生产存在薄弱环节；一些政府工作人员服务理念、工作方式跟不上发展需要，形式主义、官僚主义和消极腐败现象在一些地方和领域仍然存在。对此，我们将采取更有力的措施加以解决。

　　二、2013年工作安排

　　各位代表，2013年是全面贯彻落实十八大精神的第一年，是实施“十二五”规划承前启后的关键一年，是为全面建成小康社会奠定坚实基础的重要一年。政府工作的总体要求是：深入学习贯彻党的十八大精神，坚持以邓小平理论、“三个代表”重要思想、科学发展观为指导，紧紧围绕主题主线，以提高经济增长质量和效益为中心，稳中求进、好中求快，扎实推进新型工业化、信息化、城镇化和农业现代化，发展绿色经济，建设生态家园，加快推进“五个龙岩”建设，实现经济持续健康发展和社会和谐稳定，使闽西老区天更蓝、地更绿、水更净、人民更安康。

　　全市经济社会发展的主要预期目标是：地区生产总值增长 12%，财政总收入和地方级财政收入均增长13%；全社会固定资产投资增长22%；社会消费品零售总额增长16%；外贸出口总值增长10%；实际利用外资增长5%；城镇居民人均可支配收入增长12%，农民人均纯收入增长12%；居民消费价格总水平涨幅3.5%左右；城镇登记失业率控制在4.2%以内；人口自然增长率控制在8.7‰以内；单位生产总值能耗下降3.73%，主要污染物排放总量控制在省下达指标以内，确保实现主要经济指标增幅、城乡居民收入增幅、单位生产总值能耗降幅均高于全省平均水平。对照全面建成小康社会指标要求，逐级分解落实，逐项攻坚突破。

　　为实现上述目标，着力做好以下工作：

　　（一）加快壮大产业规模，提升经济发展质量。

　　积极发展现代农业。全面落实强农惠农富农政策，稳定粮食播种面积和产量。推进畜禽、果蔬、茶叶、林竹和水产标准化生产和品牌化经营，大力发展花卉苗木产业和林下经济，扶持发展循环农业、休闲农业、设施农业，培育“一乡一业、一村一品”，壮大一批优特农业示范基地。构建农业技术信息交流和产品展销平台，规范提升农民合作社，力争新培育产值亿元以上农业企业10家。实施烟草水源工程、44座小（二）型水库和一批山塘除险加固工程，完成农田基本建设12万亩。加强动植物重大疫病防控、防汛抗旱和森林防火，严格农产品质量安全监管。确保“三农”综合保险实现全覆盖。

　　推进工业提速增效。围绕培育"1+2"主导产业、打造"155"产业基地，抓龙头、铸链条、建集群，推进工业化与信息化、资本化融合发展。做强优势产业。做大市本级工业，突出抓好龙工配件园、汽车配件园、新龙马年产30万台汽车发动机等项目建设，推动环保装备制造向提供全方位运营、服务转型，做大产业集群。推进县域产业差异化发展，新罗区重点提升现代机械产业和服务业，永定县大力突破汽车、旅游产业，上杭县重点做强金铜产业，武平县加快发展不锈钢产业，长汀县促进稀土产业集聚发展，连城县做优新材料和旅游产业，漳平市发展特钢铸造及下游产业和茶产业。积极培育电子信息、光伏光电、生物医药、新材料、新能源等战略性新兴产业。加快改造提升。推进资源精深加工，提升钢铁、建材、煤炭产业集约化水平。抓好企业技改，推进机械、纺织、化工等产业升级，完成卓鹰、卓龙、龙化、佳丽斯等异地技改扩建。完善发展平台。进一步理顺园区管理体制，创新园区投融资机制，加强基础配套设施建设，积极发展“飞地工业”，引导产业向园区集聚，培育做大特色专业园区。加大帮扶力度。完善用电奖励、技改贴息、企业减负等政策措施，强化融资担保、市场拓展、人才、用工用地等服务，壮大实体经济。

　　全面提升服务业。完善服务业发展规划，加大政策扶持力度。全面推进中心城市和县域城区 “优二进三”，大力发展城市经济。加快一批商贸物流、城市综合体等项目建设。规划建设机械、建材、花卉、茶叶、艺术品等专业市场。扶持发展总部经济。继续实施商贸流通业“三个一百”工程，力争限上企业突破1000家。加快发展村镇银行，规范发展小额贷款公司等金融机构，防范金融风险。积极引进品牌房地产公司，加快普通商品房建设，加强房地产市场监管。加快旅游资源整合及酒店等基础设施建设，推动古田会址争创国家5A级旅游景区。加强旅游业龙头培育、品牌营销、一体运作和管理提升，抓好省级旅游综合改革试点，打造海西区域性旅游集散中心。培育发展会展经济、电子商务等新型业态，鼓励发展信息、服务外包、社区服务等服务业。

　　实施创新驱动发展战略。深入推进国家可持续发展实验区和产业示范基地建设，实施制造业信息化、科技惠民等示范工程，提升区域科技创新能力。突出企业主体，促进产学研用紧密结合。实施10个以上重大科技项目，扶持科技企业孵化器建设，积极创建工业烟尘控制技术、心脑血管新药制剂重点实验室等研发平台。完善科技创新评价标准、激励和转化机制，加强知识产权保护。

　　（二）统筹推进城乡建设，提高城镇化水平。

　　优化规划布局。围绕建设闽粤赣边区域性中心城市，抓好城市规划，科学布局中心城市“一主三新一卫”和县域城市的空间结构、功能划分。完善城市建设详规，深化综合交通、公共设施和地下空间等专项规划。注重彰显红色、客家等特色文化，突出提升重点地段、综合体、重要公建项目设计层次，严格把好质量关。加强规划审批管理，提高规划执行效力。

　　加强城乡建设。全面推进“点、线、面”攻坚计划，创建一批完整社区，确保城镇化率达到51.5%。做大做优中心城市。深入推进“城市建设年”工作，突破九大片区征迁瓶颈。加快龙岩大道三期、外环路、口岸板块、万达广场等重大项目建设。抓好污水管网、餐厨垃圾处理工程建设，加强城区人行天桥、地下通道、绿道、慢道及生态绿地规划建设。做精做美县域城镇。加强规划引导，统筹推进产业、商贸、人居和基础设施建设，完善公共服务功能。加快推进省、市小城镇综合改革建设试点和中心镇建设，打造30个工业重镇、20个商贸强镇、10个旅游名镇。建设美丽乡村。坚持统一规划、分批实施、整合资源、重点突破，抓好上杭吴地、长汀露湖、永定万石、漳平永福等美丽乡村示范点建设，打造一批集农业生产、休闲观光、文化娱乐、生态环保于一体的新农村建设精品工程。

　　提升管理水平。中心城市以“五城同创”为抓手，建设数字化城市管理系统；强化联动执法机制，继续加大违法违章建设打击力度；加快发展公共交通，新增20台以上公交车、118台以上出租车，规划建设18个公共停车场，优化路网结构，强化交通行政执法；加强物业公司管理，改善市民居住环境。县域城市以争创省级园林城市为抓手，强化城市理念，创新管理机制，推进城乡环境综合整治，提升绿化、美化水平；深入实施农村家园清洁行动，构建农村卫生保洁长效机制。逐步完善户籍、土地、住房、教育、医疗、社会保障等机制，引导农村人口向中心城市和县城、小城镇集聚。

　　加快“三区三园”建设。完善“三区三园”发展规划。进一步理顺市、县（市、区）、新区（园区）责权利关系。推进通往三个新区的快速通道建设，开工建设红尖山隧道。古蛟新区围绕推进城乡一体化发展，着力打造工业、文化旅游两大板块，加快华润希望小镇、党校迁建合建和游客服务中心等项目建设。永丰新区围绕建设生态工贸新城，突破一批重大生产性项目和新区功能配套项目。龙雁新区围绕打造国家级加工贸易梯度转移重点承接地核心区、国家级循环经济示范区，推进工业园区、人居板块和工贸综合体建设。稀土工业园区重点加快项目招商、产业聚集、做大总量，推进稀土交易市场规划建设，完善园区功能。漳平台湾农民创业园重点推进海峡茶文化交流中心等建设，发展精致农业、特色旅游业，创建美丽乡村。高新园区重点推进海西科技创业城项目前期工作，争创国家高新技术产业开发区。

　　（三）突出抓好重大项目，增强发展后劲。

　　打好“五大战役”。拓展战役范围，把现代服务业、生态建设等领域项目纳入战役组织实施，确保重点项目投资和工业投资占全社会固定资产投资的比重进一步提升。加快推进西气东输三线、海西天然气管网、赣龙铁路扩能改造工程、漳永高速公路龙岩段和冠豸山机场等项目建设；推动南三龙铁路、厦蓉高速漳州天宝至上杭蛟洋扩容工程开工建设，湖城、武平十方至城关高速公路建成通车；推进龙长高速古城互通、厦蓉高速龙岩联络线、浦建龙梅、长泉铁路等项目前期工作。

　　强化招商选资。突出招大引强，更加注重项目的科技含量、节能环保、税收贡献。加大与央企、民企、世界500强企业对接力度，力争新对接“三维”项目100个以上、总投资超过500亿元。引导异地龙岩企业家回归，落实“非禁即入”原则，鼓励社会资本进入市政公共设施和医疗、教育、养老等领域，充分激发民营经济活力。

　　完善工作机制。把项目工作列入市直部门绩效管理和效能考评的重要内容。加强投资额大、带动性强的重大生产性项目策划，重点突破机械、汽车、金铜、稀土等产业链延伸和配套项目。

　　（四）开展“生态建设年”活动，加快建设“美丽龙岩”。

　　全面推进生态市建设。认真落实市委提出的“生态建设年”部署要求，建设生态家园。总结推广“长汀经验”，继续推进以长汀、连城、永定为重点的水土流失综合治理，完成95万亩水土流失治理任务。大力实施“四绿”工程，禁止采伐天然阔叶林，加强生态公益林保护，完成造林绿化40万亩，提高森林质量。推进湿地公园等重点生态项目建设，开展绿色乡镇、绿色社区、绿色学校创建活动。力争创建省级森林城市，长汀、武平、连城、漳平创建国家级生态县（市），新罗、永定、上杭创建省级生态县（区）。落实矿区生态恢复责任，加强矿山环境综合治理。推进农村环境连片整治。建立健全生态补偿机制。

　　集约节约利用资源。全面暂停新建煤矿的勘探和审批，坚决推进新罗、永定和武平、漳平、连城小煤矿关闭重组，确保全年煤矿企业减少30家以上，提高煤炭行业集中度和标准化水平。进一步淘汰建材、冶金、化工、造纸等行业落后产能，全面淘汰中心城市落后水泥产能，严格控制排污总量。实施强制性清洁生产审核，抓好30个重点节能及循环经济项目建设。

　　抓好环境污染整治。加强水源地保护和重点流域综合治理。突出抓好龙津河流域水环境整治，按照“三年初见成效，八年全面完成”的要求，通过经济、行政、法律等手段，分期分批整治养殖业、工矿业、小水电。积极稳妥推进各县（市）主要流域畜禽养殖场综合治理。完成18条重点中小河流域治理。抓好重金属污染防治，加强化学品和危险废物管理。加大机动车尾气、餐饮业油烟、扬尘等大气污染整治力度，确保中心城市空气质量优良天数达97%以上。

　　（五）深化改革开放，增添发展动力。

　　扎实推进各项改革。继续抓好农村综合改革，创新土地流转、金融服务机制。对各县（市、区）、新区园区实施科学绩效评估和考核办法。深化行政体制改革，分类推进事业单位改革，进一步理顺市、区管理体制。推进财税管理体制改革，加强预算编制和地方政府债务管理。提升“三资”运作水平，完善投融资平台。壮大和规范担保机构。稳步推进基金、债券发行。推动龙马环卫、连城兰花等企业加快上市。深化医药卫生体制改革，抓好上杭县公立医院综合改革试点，巩固完善新农合和基本药物制度。

　　全面扩大对外开放。推动一批出口型生产企业提升扩能，培育壮大外贸出口主体。承办好第四届海峡两岸机械产业博览会，用好"6·18"、“9·8”等大型经贸合作平台。共建共享山海协作产业园区，加速融入厦漳泉大都市经济圈。推进闽西南五市、闽粤赣十三市、海西中部集中发展区的区域协作。抓好对口援藏援疆工作，加强与友好城市交流合作。

　　深化与港澳台合作。加快漳平、连城庙前台商投资区建设。推进永定客家博览园、上杭客家族谱博物馆、长汀客家首府博物馆等项目建设，办好两岸族谱论坛、旅游文化节等特色活动，提升“海峡客家”品牌。积极申报赴台个人游试点，加强海峡两岸交流基地建设，推进龙台教育、文化、科技、体育及民间交流合作。充分挖掘侨力资源，加强与香港、澳门经贸文化交流合作。

　　着力拓宽发展空间。充分发挥老区、苏区优势，重点跟进《原中央苏区振兴发展规划》，在争取国家、省重要政策、重大项目和资金支持上取得新突破。加快推进永定撤县设区，稳步推进撤乡并镇和乡改镇、村改居工作。

　　（六）保障和改善民生，维护社会和谐稳定。

　　增加城乡居民收入。抓好高校毕业生、农民工、城镇就业困难人员和退役军人就业，新增城镇就业2.2万人、农村劳动力转移就业4.5万人。落实最低工资、企业工资保证金和集体协商制度，稳步提高城乡居民特别是低收入群众收入水平。完善行政事业单位工资增长长效制度。

　　切实增强民生保障。扩大各类社会保险覆盖范围，提高城乡居民基础养老金水平和城乡医疗救助筹资标准，推进实施城乡居民大病保险和城镇居民医疗保险门诊统筹，力争城乡居民社会养老保险参保率达90%以上。加强保障性住房建设和管理，新建保障性住房5654套。统筹推进新一轮扶贫开发、农村危房改造和造福工程，抓好16个省级、30个市级集中安置区建设，完成2万人搬迁任务。解决50万人饮水安全问题。加大革命基点村和少数民族乡村扶持力度。支持发展慈善、社会福利事业，加大“老、遗、残”弱势群体帮扶力度，保障妇女儿童合法权益。加快殡葬改革。

　　提升公共服务水平。推进“教育强县”创建工作，加大义务教育标准化学校建设力度，促进教育均衡发展；加强学前教育、职业教育、继续教育工作，支持龙岩学院提升办学水平；实施“千名名教师、百名名校长”培养计划，提高教育质量。扩充医疗卫生资源总量，推进疾病防控、妇幼保健、卫生监督机构达标建设；健全农村三级医疗卫生服务网络，加强社区卫生服务中心建设。强化食品药品安全监管。实施“生育文明·幸福家庭”促进计划，综合治理出生人口性别比偏高问题，提高出生人口素质。开工建设市老年体育活动中心，办好市第十四届运动会。

　　加快发展文化事业。弘扬老区精神，践行社会主义核心价值观，提高公民思想道德素质和精神文明素养。进一步重视文化艺术工作，加快公益性文化基础设施建设。抓好古田会议旧址群等重点革命旧址维修保护，加强永定客家土楼、长汀历史文化名城等文化遗产保护开发，争创国家级闽西客家文化生态保护试验区。加强文化创意产业园建设，推进文化与旅游、创意、科技、体育、物流等产业融合发展。

　　加强和创新社会管理。深入开展“一评估四联动”，推行重大决策、重大项目社会稳定风险评估机制，健全群众利益维护机制。加强社会诚信体系建设。加快社区综合服务站建设，推行网格化管理，提升社区服务和自治功能。加强应急指挥中心建设，提高应急处置能力。深化“平安龙岩”建设，完善立体化社会治安防控体系，有效防范和坚决打击违法犯罪活动。认真落实安全生产长效机制，狠抓重点区域、重点领域整治，坚决遏制重特大安全事故。加强国防动员和后备力量建设，做好新一届省级“双拥模范城（县）”创建工作。

　　三、全面加强自身建设，打造服务型政府

　　各位代表，新的一年，各项任务十分艰巨。我们将始终坚持高效、廉洁、为民、务实，推进“红土政风”建设。

　　牢记使命，奋发有为。大力弘扬“闹革命走前头、搞生产争上游”的优良传统，解放思想，开拓创新，急争抢试，敢于突破，始终保持昂扬向上、勇于争先的创业激情。提升站位，居安思危，充分发挥老区苏区、生态环境等综合优势，用足用好中央、省赋予的扶持政策，打基础，添后劲，努力拓展龙岩发展新空间。

　　求真务实，改进作风。发扬“滴水穿石、人一我十”的精神，脚踏实地，实干实效。践行才溪乡调查精神，进一步加强调查研究，沉下身接地气，尊重群众首创精神。力戒形式主义、官僚主义，精简会议、文件、活动，讲实话、干实事，厉行节约，切实提高政府运作的效率和效益。

　　为民利民，服务至上。办好惠民实事，开展“联乡挂村帮户”和为民务实清廉教育实践活动，深入基层察民情、通民意、解民忧。推进简政放权，加强效能建设，加大治庸治懒治散力度，严格落实行政问责。

　　依法行政，清正廉洁。加强法治政府建设，自觉接受人大及其常委会的法律监督、工作监督和政协的民主监督，虚心接受舆论监督和群众监督。全面落实党风廉政建设责任制，加大对规划布局、土地出让、工程招标、拆迁安置、产权交易、民生资金使用等重点领域监管，严肃查处违法违纪案件，推进权力运行公开化、规范化，营造公平公正的社会环境。

　　各位代表，让我们紧密团结在以习近平同志为总书记的党中央周围，在市委的领导下，进一步解放思想，真抓实干，开拓奋进，为在更高起点上推进龙岩科学发展、跨越发展、加快崛起，为全面建成小康社会而努力奋斗！
